# Determinants of prenatal anemia in Ethiopia

**DOI:** 10.1186/s13690-017-0215-7

**Published:** 2017-11-06

**Authors:** Abera Abay, Haile Woldie Yalew, Amare Tariku, Ejigu Gebeye

**Affiliations:** 1 Maternal and Child Health Core Process, Asossa Zonal Health Department, Asossa, Ethiopia; 2 Department of Human Nutrition, Institute of Public Health, College of Medicine and Health Sciences, University of Gondar, Gondar, Ethiopia; 3 Department of Epidemiology and Biostatistics, Institute of Public Health, College of Medicine and Health Sciences, University of Gondar, Gondar, Ethiopia

**Keywords:** Pregnant women, Anemia, Determinants, Ethiopia

## Abstract

**Background:**

Anemia is responsible for 20% of maternal mortality worldwide, and it is associated with premature birth, low birth weight, and infant mortality. In Ethiopia, about 22% of pregnant women are anemic. However, literatures are limited, therefore, this study aimed to investigate the prevalence and associated factors of anemia among pregnant women attending antenatal care (ANC) in Asossa Zone Public Health Institutions, northwest Ethiopia.

**Methods:**

A facility based cross-sectional study was conducted from February to March 2016. Data were collected by interviewer administered, pretested and structured questionnaires. A multi-stage sampling technique was used to select 762 pregnant women. The hemoglobin level was determined by taking 5 ml of venous blood using Sahli’s method. A multivariate binary logistic regression model was fitted to identify factors associated with anemia. Adjusted Odds Ratio (AOR) with a 95% Confidence Interval (CI) was computed to show the strength of association and statistical significance was determined at a *P*-value of <0.05.

**Results:**

The prevalence of anemia was 31.8% [95% CI: 28.9, 35.5]. In the adjusted analysis, maternal age of 30–34 years [AOR = 0.34, 95% CI: 0.14, 0.86], household size of ≥6 [AOR = 4.27, 95% CI: 1.58, 11.45], dietary diversity [AOR = 0.58, 95% CI: 0.38, 0.93], no meat consumption [AOR = 1.80, 95% CI: 1.11, 2.91], not drinking soft beverages [AOR =1.96, 95% CI: 1.19, 3.23], undernutrition [AOR = 7.38, 95% CI: 4.22, 12.91], not consuming fruits [AOR = 3.29, 95% CI: 1.59, 6.82], inter-pregnancy interval of ≥2 years [AOR = 0.59, 95% CI: 0.34, 0.99], and third trimester of pregnancy [AOR = 0.33, 95% CI: 0.20, 0.57] were significantly associated with anemia.

**Conclusions:**

The prevalence of prenatal anemia is high in the Asossa Zone; suggesting a moderate public health concern. Socio-demographic and dietary intake characteristics were significantly associated with anemia. Therefore, improving dietary diversity and animal food consumption are the key to reduce the high burden of anemia. It is also important to strengthen interventions aiming to reduce closed birth interval and teenage pregnancy.

## Background

Anemia is a nutritional disorder resulted from a hemoglobin level below the established normal reference values. It exists as mild to moderate public health problem in developed and developing countries [1]. Despite anemia affects all segments of the population, pregnant women are the most vulnerable groups because of their unique physiological state. Anemia is correlated with adverse health consequences and affects the socio-economic development of the country [2, 3]. Worldwide, anemia is an important preventable cause of maternal and perinatal morbidity and mortality [4]. It is causes 20% of maternal death and associated with premature birth, low birth weight, and infant mortality. Moreover, it impairs the growth and learning ability of children, resistance to infections, and physical work capacity and productivity of adults [5–7].

Globally, about 38.2% of pregnant mothers are anemic, while almost two-third are anemic in developing countries. In Africa, prenatal anemia was detected in 48.7% of mothers [8]. Ethiopian has been undertaking different measures (iron-folate supplementation, malaria treatment and control strategies, and deworming) to control anemia [7]. However, the 2011 Ethiopian Demographic and Health Survey report showed that, still 22% of pregnant mothers are suffering from anemia [9].

The former researches in different developing countries, including Ethiopia, illustrated that maternal anemia is multi-factorial. Accordingly, age, place of residence, marital status, employment status, household size, educational and wealth status are socio demographic and economic determinants of anemia. Chronic energy deficiency, meal frequency, dietary diversity, gravidity, parity, inter-pregnancy interval, gestational age and history of infectious disease, malarial attack and intestinal parasitic infestation are also significantly associated with anemia in pregnant mothers [8–24].

Obviously, monitoring of health problems and its determinants is essential for developing effective interventions [19]. Particularly, it has a special importance for countries, like Ethiopia where the burden of health and nutritional problems, including amenia is high [10]. However, there is limited scientific evidence, especially in Benishangul Gumz Regional State, the study area. Thus, this study aimed to assess the prevalence and associated factors of anemia among pregnant mothers attending ANC clinic in public health institutions of Asossa Zone, northwest Ethiopia.

## Methods

### Study design and settings

A facility-based cross-sectional study was conducted from February to March 2016 in Asosa Zone which is found in Benishangul Gumuz Regional State, northwest Ethiopia. The zone lies at 580–1668 m above sea level and 661 km far from the capital of Ethiopia, Addis Ababa. According to the recent (2015/2016) Regional Finance and Economic Development Office projection, total population of the zone was estimated at 377852, in which 12,885 were pregnant women. Furthermore, the health coverage was 86%, and 11 public health centers and one general hospital were providing health service to the community during the data collection time [25].

### Study population and sampling procedure

All pregnant mothers attending ANC clinic for their regular follow-up in Asossa Zone Public Health Institutions during the study period were eligible for the study. A single population proportion formula was used to estimate sample size. Assumptions, including prevalence of anemia in Benishangul Gumuz Regional State as 28% [12], 95% confidence level, 4% margin of error, 5% non-response rate, and a design effect of 1.5 were considered to obtain sample size of 762.

A multi-stage sampling technique was employed to select the study participants. The public health facilities were stratified into health centers and hospital, and a lottery method was used to choose 5 health facilities (4 health centers and 1 general hospital) from the total. Quantity of pregnant mothers attended (1676) ANC in the previous year (2015) in the selected health institutions was taken from the registration log book to estimate sampling fraction. Total samples included in each health facility were proportionate to population size and then systematic sampling technique was employed using the calculated sampling interval (k^th^ = 1676/762 = 2.2) to select the study participants.

### Data collection instrument and procedures

Pretested interviewer-administered questionnaire was used to collect data. The English version questionnaire was translated into Amharic language (native language of the study area) and then back translated to English by language and public health experts. A total of 13 data collectors (6 nurses and 5 laboratory technicians) and 2 public health officers as supervisor were recruited for the study.

A 5 ml of venous blood was collected into 2/3 of micro-hematocrit tube with anti-coagulant and centrifuged for 5 min. Hemoglobin estimation was done by comparative Sahli’s method and the result was expressed in g/dl. Hemoglobin value was adjusted by considering altitude of the study area [26]. Finally, severity of anemia was defined as non-anemic (hemoglobin level ≥ 11.0 g/dl), mild anemia (hemoglobin level of 10.0–10.9 g /dl), moderate anemia (hemoglobin level of 7.0–9.9 g/dl), and severe anemia (hemoglobin level of <7 g/dl) [27].

Nutritional status of participants was assessed by measuring the Mid-Upper Arm Circumference (MUAC) using the measuring tape. Accordingly, undernutrition was ascertained when the MUAC measurement was ≤21 cm [28].

Dietary Diversity Score (DDS) of pregnant women was calculated by using a 24–hour recall method. An open recall method was employed to gather information about the foods and drinks consumed by the study participants. Accordingly, woman was requested to list what she ate in the past 24 h prior to the date of interview. The score was computed based on 9 food groups which aimed to reflect the micronutrient adequacy of the diet. Finally, mother’s dietary intake was categorized into poor, medium and high dietary diversity score if she consumed ≤3 food groups, 4–5 food groups and ≥6 food groups, respectively [29].

Livestock ownership, selected household assets, size of agricultural land, and the quantity of crop production were the variables used in determining the household wealth status. A principal components analysis was used and the factor scores were summarized into terciles (poor, medium and rich) [30].

Three days of training was given to data collectors and supervisors. The training mainly focused on the purpose of the study, techniques of interview, and important ethical issues of the research project. Pretest was done on 5% of the total sample out of the study area, Abrhamo Health Center. During pre-test, the applicability of data collection procedures and tools were evaluated. Regularly all questioners were checked for completeness, clarity and consistency by the supervisors and investigators.

### Data processing and analysis

Data were entered into EPI INFO version 7 and analyzed using the Statistical Package for Social Sciences (SPSS) version 20. Descriptive statistics, including frequencies and proportions were used to summarize variables. A binary logistic regression model was used to identify factors associated with anemia. Variables with a *P*-value of <0.2 in the bivariate analysis were exported to the multivariate analysis to control the possible effect of confounders. The Adjusted Odds Ratio (AOR) with a 95% confidence level was estimated to show the strength of association, and a *P*-Value of <0.05 was used to declare the statistical significance in the multivariate analysis.

## Results

### Socio-demographic and economic characteristics

A total of 761 pregnant mothers were participated in this study giving a response rate of 99.9%. The median age was 25.0 year with an inter-quartile range of 7.0 year. Three-fourth (76.5%) of the study participants were urban inhabitants (Table 1).

**Table 1:** Socio–demographic and economic characteristics of pregnant mothers attending antenatal care clinic in Asossa Zone Public Health Institutions, northwest Ethiopia, 2016 (*n* = 761)

Characteristics	Frequency	Percent
Age (in years)		
< 20	76	10.0
20–24	247	32.5
25–29	264	34.7
30–34	126	16.6
≥ 35	48	6.3
Religion		
Muslims	368	48.4
Orthodox	301	39.6
Protestant	92	12.0
Place of residence		
Urban	582	76.5
Rural	179	23.5
Ethnicity		
Berta	215	28.3
Shinasha	56	7.4
Amhara	256	33.6
Oromo	164	21.6
Tigre	43	5.7
Others	27	3.5
Marital status		
Married	727	95.5
Unmarried^a^	34	4.5
Educational status		
No formal education	333	43.8
Primary education	107	14.1
Secondary education	123	16.2
Certificate and above	198	26.0
Employment status		
House wife	503	66.1
Government employee	194	25.5
Self employed	46	6.0
Others	18	2.4
Family size		
≤ 2	252	33.1
3–5	398	52.3
≥ 6	111	14.6
Household wealth status		
Poor	208	27.4
Medium	319	41.9
Rich	234	30.7

^a^single, divorced and widowed

### Prevalence of anemia, dietary habit, and health related characteristics

More than half (56.8%) of the study participants had 3 meals per a day. About 62.2% of study subjects ate meat at least once per week. One-quarter (25.4%) of women had high DDS and about 85.5% were well nourished (Table 2). Half (45.9%) of the pregnant mothers were found at third trimester of pregnancy. Majority of (77.1%) the study participants had history of closed inter-pregnancy interval (less than two years) and 13.4% had history of repeated malaria infection (Table 3).

**Table 2:** Dietary pattern and nutritional status related characteristics of pregnant mothers attending antenatal care clinic in Asossa Zone Public Health Institutions, northwest Ethiopia, 2016 (*n* = 761)

Characteristics	Frequency	Percent
Meal frequency per day		
≥ 4 times	237	31.1
3 times	432	56.8
≤ 2 times	92	12.1
Frequency of meat consumption per week		
≥ Once per week	473	62.2
None	288	37.8
Tea/coffee consumption per day		
Yes	726	95.4
No	35	4.6
Consumption of soft drinks per week		
≥ Once per week	544	71.5
Never	217	28.5
Fruit consumption per week		
Greater than twice per week	183	24.0
Twice per week	260	34.2
Once per week	196	25.8
Once per month	122	16.0
Egg consumption per week		
Every day	23	3.0
Once per week	230	30.2
More than or equal to twice per week	137	18.0
Once per month	154	20.2
None	217	28.5
Milk and milk products consumption per week		
More than once per day	25	3.3
Once per day	75	9.9
Once per week	320	42.0
None	341	44.8
Staple food of the family		
Injera	621	81.6
Porridge	140	18.4
Dietary Diversity Scores		
Poor	191	25.1
Medium	377	49.5
High	193	25.4
MUAC Measurement		
≤ 21 cm	110	14.5
≥ 22 cm	651	85.5

**Table 3:** Health care related characteristics of pregnant women attending antenatal care clinic in Asossa Zone Public Health Institutions, northwest Ethiopia, 2016 (*n* = 761)

Variables	Frequency	Percent
Gravidity of the mother		
One	255	33.5
Two	212	27.9
Three and above	294	38.6
Parity of the mother		
Null–parous	260	34.2
Para–One and Two	212	27.9
Para–Three and above	289	38.0
Gestational age		
First trimester	154	20.2
Second trimester	258	33.9
Third trimester	349	45.9
Inter pregnancy interval in years		
< 2	587	77.1
≥ 2	174	22.9
History of abortion		
Yes	48	6.3
No	713	93.7
History of repeated malaria infection		
Yes	102	13.4
No	659	86.6
History of chronic illness		
Yes	19	2.5
No	742	97.5
Intestinal parasite infestation in past one week		
Yes	7	1.0
No	754	99.0
Iron- folate supplementation		
Yes	647	85.0
No	114	15.0

**Fig. 1:**
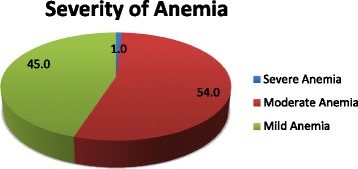
Severity of anemia among pregnant women attending antenatal care at Asossa Zone Public Health Institutions, northwest Ethiopia, 2016 (*n* = 761)

About 31.8% [95% CI: 28.9, 35.5] of pregnant mothers were anemic in the study area, of which 54.0% were moderately anemic. After adjustments were done for altitude, the median hemoglobin level was 11.80 g/dl and the Inter-Quartile Range was 2.0 g/dl (Fig. 1).

### Factors associated with anemia

In the multivariate logistic regression analysis, age of the mother, household size, meat, soft drink and fruit consumption, DDS, nutritional status, gestational age and inter-pregnancy interval were independently and significantly associated with anemia.

In this study, the odds of developing anemia were 4.27 higher among mother who belonged to a household size of ≥6 as compared to mothers living in a household size of ≤2 [AOR = 4.27, 95 % CI: 1.58, 11.45]. The likelihood of having anemia was 80% higher among mothers who did not eat meat in the past 1-week (prior to the date of survey) compared to those who ate at least once per week [AOR = 1.80, 95 % CI: 1.11, 2.91].

The odds of anemia were 66% less in mothers aged 25–29 years when compared to those aged <20 years [AOR = 0.34, 95% CI: 0.14, 0.86]. Also, the lesser odds of anemia were detected in mothers who had DDS of 4–5 [AOR = 0.58, 95% CI: 0.38, 0.93] and inter pregnancy interval of ≥2 years [AOR = 0.59, 95% CI: 0.34, 0.99]. Similarly, the odds of having anemia were 67% less among mothers found in the third trimester of pregnancy compared to those found in the first trimester of pregnancy [AOR = 0.33, 95% CI: 0.20, 0.57].

Soft drink consumption was also significantly associated with anemia. The odds of developing anemia were near to two times higher among mothers without history of weekly soft drink consumption compared to their counterparts [AOR =1.96, 95% CI: 1.19, 3.23]. Those pregnant mothers who did not consume fruit at least once per week were found at higher odds of developing anemia [AOR = 3.29, 95% CI: 1.59, 6.82]. Finally, the likelihood of anemia was 7.38 times higher in undernourished mothers (MUAC ≤21 cm) compared to well-nourished ones [AOR = 7.38, 95% CI: 4.22,12.91] (Table 4).

**Table 4:** Factors associated with anemia among pregnant mothers attending antenatal care clinic in Asossa Zone Public Health Institutions, northwest Ethiopia, 2016 (*n* = 761)

Variables	Anemia		COR (95% CI)	AOR (95%CI)
	Yes (#)	No (#)		
Mother age in years				
< 20	30	46	1.00	1.00
20–24	72	175	0.63 (0.37,1.08)	0.63 (0.31,1.27)
25–29	73	191	0.59 (0.344, 0.99)	0.39 (0.18, 0.85)^*^
30–34	43	83	0.79 (0.44, 1.43)	0.34 (0.14, 0.86)^*^
≥ 35	24	24	1.53 (0.74, 3.18)	0.47(0.15,1.49)
Educational status				
No formal education	139	194	2.50 (1.68, 3.74)	0.67 (0.32, 1.40)
Primary education (1–8)	33	74	1.56 (0.92, 2.65)	0.93 (0.42, 2.03)
Secondary education (9–12)	26	97	0.94 (0.54, 1.62)	0.98 (0.46, 2.10)
Certificate and above	44	154	1.00	1.00
Employment status				
House wife	179	324	1.00	1.00
Government employee	49	145	0.61 (0.42, 0.89)	0.93 (0.48, 1.82)
Self employed	12	34	0.64 (0.32, 1.23)	1.63 (0.72, 3.73)
Others	2	16	0.23 (0.05, 0.99)	0.78 (0.16, 3.88)
Marital status				
Currently married	226	501	1.00	1.00
Currently unmarried	16	18	1.97(0.99, 3.94)	2.25 (0.94, 5.36)
Residence				
Urban	158	424	1.00	1.00
Rural	84	95	2.37(1.68, 3.35)	0.66 (0.33,1.30)
Household size				
≤ 2	52	200	1.00	1.00
3–5	132	266	1.91(1.32, 2.76)	2.30 (1.06, 4.99)^*^
≥ 6	58	53	4.21(2.60, 6.81)	4.27(1.58,11.50)^**^
Household Wealth status				
Poor	80	128	1.00	1.00
Medium	81	238	0.55 (0.37, 0.79)	0.77 (0.47,1.28)
Rich	81	153	0.85 (0.58,1.25)	1.44 (0.79, 2.62)
Meal frequency per day				
≥ 4 Times	53	184	1.00	1.00
3 Times	144	288	1.74 (1.21, 2.50)	0.68 (0.42, 1.11)
2 Times	47	45	3.32 (2.00, 5.54)	0.94 (0.45, 1.96)
Meat consumption per week				
≥ Once per week	114	359	1.00	1.00
None	128	160	2.52 (1.84, 3.45)	1.80 (1.11, 2.91)^*^
Drinking of soft beverages per week				
≥ Once per week	133	411	1.00	1.00
None	109	108	3.12 (2.24, 4.34)	1.96 (1.19, 3.23)^**^
Fruit consumption per week				
> Twice per week	30	153	1.00	1.00
Twice per week	57	203	1.43 (0.88, 2.34)	1.01 (0.57, 1.79)
Once per week	72	124	2.96 (1.82, 4.82)	2.14 (1.20, 3.81)^*^
None	83	39	10.9 (6.29,18.73)	3.29 (1.59, 6.82)^**^
Egg consumption per week				
Every day	3	20	1.00	1.00
Once per week	62	168	2.46 (0.71, 8.57)	2.43 (0.63, 9.34)
≥ Twice per week	26	111	1.56 (0.43, 5.65)	1.73 (0.43, 6.87)
Once per month	77	77	6.67(1.90, 23.36)	2.35 (0.58, 9.54)
Never	74	143	3.45 (0.99,11.99)	1.44 (0.36, 5.80)
Staple foods of the family				
Injera	180	441	1.00	1.00
Porridge	62	78	1.95 (1.34, 2.84)	1.33 (0.71, 2.49)
Dietary Diversity Score				
Poor	89	102	1.00	1.00
Medium	94	283	0.38 (0.26, 0.55)	0.58 (0.36, 0.93)^*^
High	59	134	0.51(0.33, 0.77)	1.03 (0.59, 1.80)
MUAC measurement				
≤ 21 cm	81	29	8.50 (5.37,13.47)	7.38 (4.20, 12.90)^***^
≥ 22 cm	161	490	1.00	1.00
Gravidity of the mother				
Gravida–One	58	197	1.00	1.00
Gravida–Two	70	142	1.67(1.11, 2.52)	0.46 (0.07, 2.79)
Gravida–Three and above	114	180	2.15 (1.48, 3.13)	0.51(0.06, 4.54)
Parity of the mother				
Null–Parous	57	203	1.00	1.00
Para–One to Two	72	140	1.83(1.22, 2.76)	3.98 (0.61, 25.98)
Para–Three and above	113	176	2.29 (1.57, 3.33)	3.54 (0.37, 34.25)
Gestational age in (trimester)				
1st Trimester	70	84	1.00	1.00
2nd Trimester	88	170	0.62 (0.41, 0.94)	0.61(0.36, 1.04)
3rd Trimester	84	265	0.38 (0.23, 0.57)	0.33 (0.2, 0.57)^***^
Birth interval in (years)				
< 2	201	386	1.00	1.00
≥ 2	41	133	0.59 (0.40, 0.87)	0.59 (0.34, 0.99)^*^
History of repeated malaria infection				
Yes	43	59	1.00	1.00
No	199	460	0.59 (0.39, 0.91)	1.19 (0.68,2.09)

Note:^*^ *P–value <0.05;* ^**^ *P–value <0.01;* ^***^ *P –value <0.00*

## Discussion

The burden of prenatal anemia is widely recognized as a major public health problem throughout the world, particularly in developing countries [8]. Because of blood volume expansion and increased iron demand of the fetus and the mother, hemoglobin level altered dramatically during the course of pregnancy [27].

This study noted that, the prevalence of anemia was 31.8% which confirmed the moderate public health significance of the problem. This finding was comparable with the former study from Ethiopia (27.8%) [13] and other developing countries, such as, Brazil (28.1%) [23] and Uganda (29.1%) [19]. The result was slightly higher than the previous local reports (16.6–22%) [9, 12, 31]. However, this report was lower than another study from Ethiopia (53.9%) [32] and Nigeria (54.5%) [33], Ghana (57.1%) [20], Burkina Faso (61%) [34] and Uganda (63.1%) [35]. The variation in the burden of anemia between the current and latter study settings could be related to disparities in occurrence of malaria and hookworm infestation. An increased magnitude of malaria [32, 34] and other febrile illness [33] and intestinal parasitic infestation [20, 32] are reported in the former studies. Febrile illnesses, including malaria, and parasitic infestation are correlated with reduced blood hemoglobin level [20, 32, 33]. Furthermore, higher utilization of iron-folate supplementation might explain the lower prevalence of anemia in the study area compared to what was reported in Nigeria [33] and Burkina Faso [34].

The result of multivariate analysis showed that, mothers age was significantly associated with anemia; the likelihood of developing anemia was lower among women aged 25–29 and 30–34 years compared to those aged <20 years. Similar findings were also reported by other studies, for instance Ethiopia [36], Uganda [18], Ghana [20], Thailand [37], and Turkey [38]. A former study also demonstrated that anemia is the common nutritional problem in teenage pregnancy [39]. Obviously, adolescence (10–19 years) is a state of rapid growth and development [40] which ultimately increases iron requirement (2.2 mg iron/day) by a fold compared to the preadolescent period (6–9 years) (0.7–0.9 mg iron/day) [41]. Unfortunately, if an adolescent girl becomes pregnant, the mother and fetus will compute for nutrients to support their rapid growth which in turn increases her vulnerability for anemia [42, 43].

Similarly, the higher likelihood of developing anemia was noted among mothers from the larger (≥6) family size compared to those from a smaller (≤2) family size. This finding was supported by the previous reports elsewhere; Ethiopia [12], Brazil [23] and India [44]. Most of the time large family size is associated with low socio-economic status of the household, for that reason little resources may be available to nourish the entire family members. Additionally, large family size is a strong indicator for closed birth spacing, which in turn affects maternal hemoglobin status [45].

Lack of meat consumption in the previous 1-week was significantly associated with higher odds prenatal anemia. This report was consistent with the reports from Ethiopia [15, 46], Pakistan [8], Turkey [38] and Vietnam [47]. Meat is a rich source of hem-iron which has better bioavailability compared to non-heme-iron, form of iron majorly obtained from plant based food groups [15, 48], On the other hand, hem-iron enhances absorbability of non-hem iron [49].

Furthermore, vitamin–C, a chief reducing equivalent, enhances absorption of iron in the gastrointestinal mucosa [50]. Given that, poor consumption of Vitamin-C rich food (fruits and vegetables) increases risk of developing anemia. In line to this fact, this study showed that the odds of anemia were increased among mothers who had no fruit intake in the previous week prior to date of data collection. Parallel findings were also reported from Pakistan [8] and Turkey [38].

This study also noted that the likelihood of having anemia was lower among mothers with diversified diet. Similar results were also reported from Ethiopia [31], Pakistan [8], and Turkey [38]. Dietary diversification, a proxy indicator of micronutrient adequacy of the diet, has special importance for the countries, like Ethiopia, in which the dietary habit of the population is relied on the monotonous cereal based food [51]. Cereals are energy dense, but poor in micronutrients.

This study illustrated that, drinking of soft beverages at least once per week was associated with reduced odds anemia. The result was in agreement with the earlier studies [35, 52]. Despite acidic beverages improve iron absorption, some of beverages containing tannin and caffeine are known to inhibit iron absorption [32]. Therefore, the relationship between soft drinks and anemia needs further investigation.

The odds developing anemia were higher among undernourished (MUAC ≤ 21 cm) mothers. The finding was supported by studies done in developing countries, such as Ethiopia [14], Kenya [53], India [54], and Nepal [55]. Pregnancy is the most nutritionally demanding time in a woman’s life, which increases the vulnerability of mothers for poor micronutrient reserve, including iron [51]. In addition, undernutrition impaired production of iron transport proteins and increased depletion of stored iron which in turn causes anemia [32, 56].

The risk of maternal nutritional depletion also increases with closed birth intervals and repeated pregnancies. Therefore, mothers need adequate time to restore nutritional reserve until the next pregnancy [57]. Mothers attain good nutritional status, including iron, when there is a gap of at least 2 years between consecutive pregnancies [58, 59]. In line to this fact, this study showed lower odds of developing anemia among women with greater than or equal to 2 years of inter-pregnancy interval compared to their counterparts. The finding was consistent with the previous studies in Ethiopia [60], Nepal [24] and India [61, 62].

The lower odds of anemia were also detected in the third trimester of pregnancy compared to those who were in the first trimester of pregnancy. However, it is not in line with the former local [13–15, 31, 63] and abroad reports of developing countries [8, 64]. Obviously, the risk of anemia increases with advancement of trimester of pregnancy [60]. Hemoglobin concentration starts declining during first trimester and reaches to lowest level during second trimester and rises again at the third trimester of pregnancy [28]. This might explain the lower odds of anemia in the third trimester of pregnancy in the current study. Moreover, most of the pregnant women starts to attend antenatal care in the second trimester of pregnancy and iron-folate supplementation is also given which in turn reduces mother’s nutritional depletion for iron.

This study investigated the burden of anaemia in the wider study area. However, some of the limitations ought to be taken into account. Cross–sectional nature of this study may not show temporal relationship between the dependent and independent variables. Moreover, ascertainment of repeated malaria infection was relied on the memory/information given by study participants, which might be subjected to recall bias.

## Conclusions

The study revealed that, the burden of prenatal anemia is high and exists as moderate public health concern in Assosa Zone. In addition, prenatal anemia was majorly associated with socio-demographic and nutrition related factors. Therefore, improving dietary diversity, meat, energy, and fruit consumption are critical to mitigate maternal anemia. It is also important to strengthen measures aiming to address early pregnancy and closed birth interval.

## Abbreviations

ANC: Antenatal care
AOR: Adjusted odds ratio
CI: Confidence interval
COR: Crude odds ratio
DDS: Dietary Diversity Score
MUAC: Mid-Upper Arm Circumference
SD: Standard deviation
SPSS: Statistical Package for Social Sciences
